# Cardiac implantable electronic device carriers undergoing transcatheter tricuspid valve annuloplasty: real-world insights

**DOI:** 10.1007/s00392-025-02616-5

**Published:** 2025-03-10

**Authors:** Jan M. Wrobel, Johannes Kirchner, Kai Friedrichs, Thorsten Gietzen, Jan Althoff, Caroline Hasse, Philipp von Stein, Jonas Wörmann, Jennifer von Stein, Jonathan Curio, Felix Rudolph, Maria Ivannikova, Christos Iliadis, Daniel Steven, Stephan Baldus, Volker Rudolph, Roman Pfister, Muhammed Gerçek, Maria I. Koerber

**Affiliations:** 1 Heart Center, Department III of Internal Medicine, Faculty of Medicine and University Hospital Cologne, University of Cologne, Kerpener Straße 62, 50937 Cologne, Germany; 2 Clinic for General and Interventional Cardiology/Angiology, Herz- Und Diabeteszentrum NRW, Ruhr-Universität Bochum, Med. Fakultät OWL (Universität Bielefeld), Bad Oeynhausen, Germany

**Keywords:** Tricuspid regurgitation, Transcatheter tricuspid valve annuloplasty, Cardiac implantable electronic devices, TVARC, Efficacy, Safety

## Abstract

**Background:**

Transtricuspid cardiac implantable electronic devices (CIEDs) complicate the management of tricuspid regurgitation (TR). Transcatheter tricuspid valve annuloplasty (TTVA) offers a promising approach due to minimal interaction with leaflets and transvalvular CIEDs, though real-world evidence is limited.

**Methods:**

This bi-center, retrospective study includes 204 consecutive patients who underwent TTVA with the Cardioband (Edwards Lifesciences) for severe symptomatic TR. Patients were divided into CIED carriers and non-CIED carriers. CIED carriers were further classified into those with lead-associated TR (LTR-A) and those with TR unrelated to CIED leads (LTR-B).

**Results:**

Among the 204 patients, 41 (20%) were CIED carriers. Of these, 24% had mixed TR etiology (functional and LTR-A), while 76% had predominantly functional TR (LTR-B). Compared to non-CIED-carriers, CIED carriers were more symptomatic (NYHA-FC > II; 93% vs. 89%; *p* = 0.026) with comparable TR severity at baseline. Intraprocedural success according to the Tricuspid Valve Academic Research Consortium was 68% in CIED carriers and 70% in non-CIED carriers (*p* = 0.851). LTR-A was associated with poorer TR reduction immediately after TTVA (*p* = 0.022). Overall safety was comparable, with right ventricular lead dislodgement occurring in one patient. Beyond that, CIED function remained unimpaired.

At 30 days, echocardiographic follow-up showed comparable TR reduction (TR ≤ II: 56% vs. 68%; *p* = 0.219) and NYHA-FU ≤ II (63% vs. 70%; *p* = 0.524) in CIED-and non-CIED carriers, respectively.

**Conclusions:**

TTVA achieves significant TR reduction, providing a safe and effective therapeutic option for TR treatment in CIED carriers.

**What is known?**

TTVA using the Cardioband has been approved for severe, symptomatic TR patients, however data on the safety and efficacy in CIED carriers is lacking.

**What the study adds?**

- Intraprocedural success and safety were comparable in CIED and non-CIED carriers treated with TTVA.
- Subgroup analyses showed a trend towards worse outcome and efficiency of TTVA in patients with LTR-A.
- Postinterventional CIED interrogations did not show critical technical issues.

**Graphical abstract:**

Intraprocedural success and TR reduction following TTVA in CIED- and non-CIED-carriers. Blue arrows indicate CIED lead trajectory through tricuspid valve.

**Figure:**
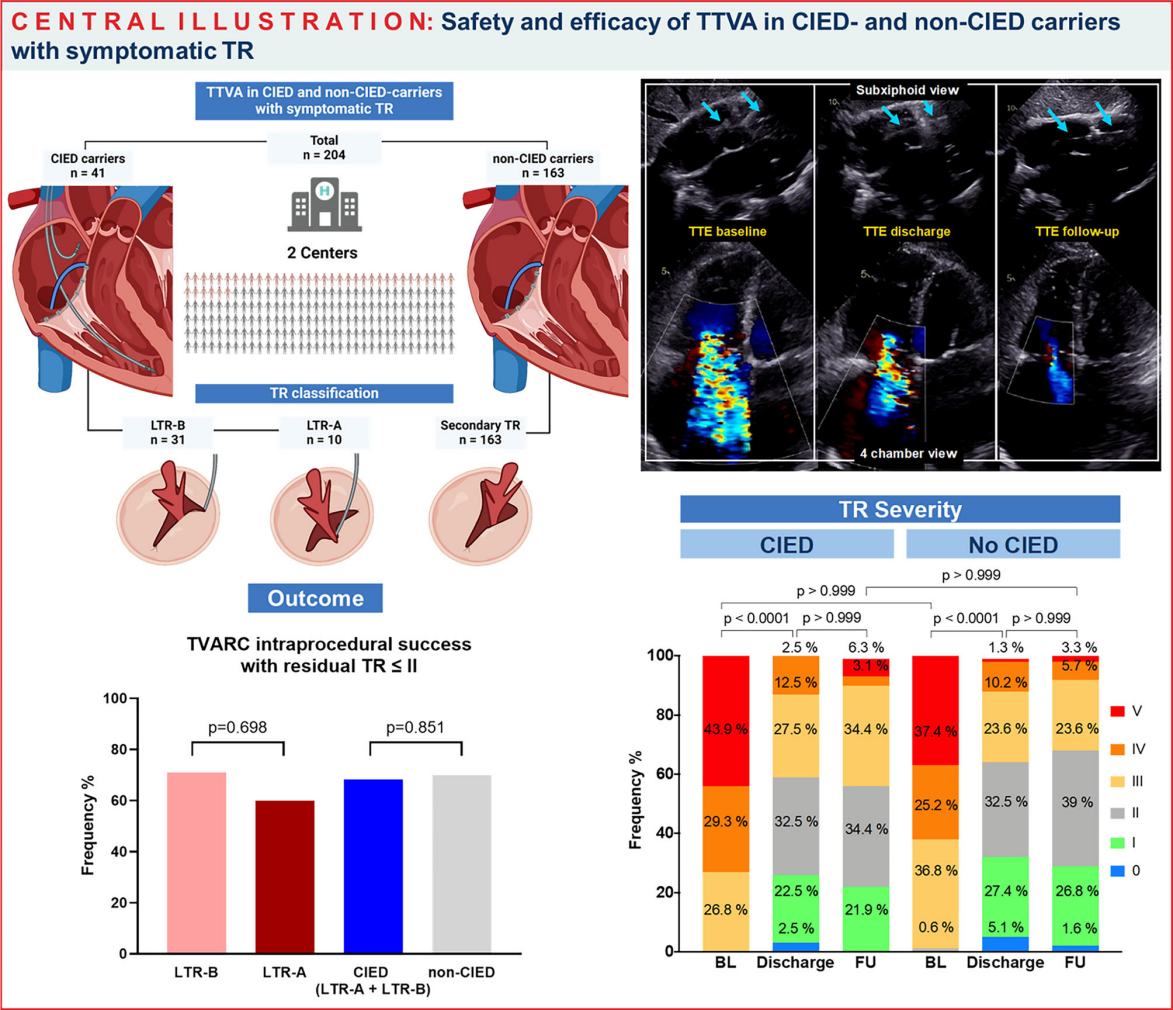
Safety and efficacy of TTVA in CIED- and non-CIED carriers with symptomatic TR

*BL* Baseline, *FU* = 30-day Follow Up, *LTR-A*  Lead-associated Tricuspid Regurgitation, type A, *LTR-B*  Lead-associated Tricuspid Regurgitation, type B, *TR* Tricuspid Regurgitation, *TTVA* Transcatheter Tricuspid Valve Annuloplasty

**Supplementary Information:**

The online version contains supplementary material available at 10.1007/s00392-025-02616-5.

## Introduction

Tricuspid regurgitation (TR) is associated with poor outcomes, with its prevalence rising to 4% in those over 75 years of age and up to 45% following cardiac implantable electronic device (CIED) implantation, with CIED-related TR accounting for 10–15% of all cases [1, 3, 26]. This issue is expected to grow due to aging demographics and increased CIED use. Lead-associated TR (LTR), categorized into type A (lead interference, 14% of LTR cases) and B (no causative link of TR to leads), can complicate the treatment approach [5]. Isolated lead extraction often fails to fully address TR due to secondary atrial or ventricular remodeling and is further complicated by continuous stimulation dependency and pronounced frailty. Conversely, isolated tricuspid valve surgery is associated with a high in-hospital mortality of up to 12%, prompting exploration of lower risk treatment alternatives [8]. Consequently, transcatheter interventions, such as direct tricuspid valve annuloplasty (TTVA), are actively being pursued to treat severe TR in this frail patient collective. Despite the potential of TTVA and other transcatheter techniques, such as tricuspid transcatheter edge-to-edge repair (T-TEER) and transcatheter tricuspid valve replacement (TTVR), earlier trials have largely underreported CIED patients [4, 7, 9, 15, 17, 19]. TTVA is considered suitable for patients with RV leads, avoiding leaflet grasping issues seen with T-TEER. However, the lack of comprehensive, real-world data on the outcomes and risks associated with TTVA in the CIED population is a notable concern. In the present work, we evaluated the safety and efficacy of TTVA in CIED-carriers.

## Methods

### Study population

This retrospective, bi-center analysis includes consecutive patients with severe, symptomatic TR receiving TTVA with the Cardioband (Edwards Lifesciences, Irvine, CA, USA) between October 2018 and September 2023 at 2 high-volume centers in Germany (Heart Center at the University Hospital of Cologne and Heart and Diabetes Center North Rhine-Westphalia in Bad Oeynhausen). Outcomes following TTVA were assessed in patients with and without CIEDs, termed CIED-carriers and non-CIED-carriers, respectively. Only CIED carriers with a transtricuspid lead were considered. Patients with leadless pacemakers were excluded. The individual decision to perform TTVA or T-TEER was guided by tricuspid leaflet morphology, coaptation gap, and CIED lead positioning. TTVA was preferred for patients with wide coaptation gaps, severe leaflet tethering or thickening, bileaflet tethering, large annulus size, or complex coaptation planes. Additionally, centrally positioned transtricuspid CIED leads that could hinder clip deployment by interacting with the leaflet grasping zone favored TTVA. In contrast, T-TEER was considered for patients with small coaptation gaps, simple leaflet morphology, and leads traversing the posteroseptal commissure without signs of lead impingement. Patients were deemed inoperable or at prohibitive surgical risk by an interdisciplinary heart team, with procedural feasibility confirmed by the device manufacturer following comprehensive imaging including cardiac computer tomography.

### Data assessment

Data were collected from electronic health records, following institutional review board approval. No informed consent was required. Anonymized data were analyzed centrally. Transthoracic echocardiography (TTE) at baseline, discharge, and 30 days post-TTVA was locally assessed per current recommendations [18]. TR severity was evaluated at baseline, after device placement, at discharge, and at a 30-day follow-up, based on the classification by Hahn et al. [14]. Clinical follow-up data were obtained during routine outpatient visits or via phone contact with patients or their general practitioners.

LTR was classified according to the Tricuspid Valve Academic Research Consortium (TVARC) into LTR-A (CIED lead contributing to TR with leaflet impingement or restrictions) and LTR-B (incidental CIED leads unrelated to TR) [13]. In a transgastric view, lead impingement/restrictions were defined as leaflet interaction restricting leaflet movement and worsening TR. Procedural guidance and RV lead position assessment used three-dimensional transesophageal echocardiography (TEE), following recommendations by Addetia and colleagues [1]. Electrocardiogram (ECG)-triggered cardiac computer tomography (CT) scans aided procedural planning. Digital patient files provided postprocedural event information. Pre- and post-procedural CIED interrogations (within 6 months pre-intervention and 6 weeks post-TTVA) were reviewed for device functionality.

### Procedure

All procedures were performed under general anesthesia with TEE and fluoroscopic guidance. The implantation technique for the CE-approved Cardioband® system has been outlined in detail previously [17, 22].

### Endpoints

Intraprocedural success, the primary efficacy endpoint, was defined by TVARC, as successful device deployment and reduction of TR to mild or moderate in post-device implantation TEE, in the absence of procedure-related complications, emergency surgery (including pericardiocentisis) or readmissions for the underlying condition within 30 days [13].

Symptomatic changes were assessed using the New York Heart Association functional class (NYHA-FC), and NTproBNP levels and diuretic therapy were assessed at baseline and 30-day follow-up. TVARC safety endpoints included 30-day all-cause mortality, acute kidney injury (AKI) stage 4, life-threatening bleeding (type 5), heart failure hospitalization, conduction disturbances, and specific device-related complications. Peri-interventional CIED monitoring followed European Heart Rhythm Association (EHRA) Expert Consensus [28]. A one-year subgroup analysis of overall survival and heart failure re-hospitalization was conducted between LTR-A, LTR-B and non-CIED carriers.

### Statistical analysis

Categorical variables were presented as percentages, and continuous variables as means with standard deviations (SD) or medians with interquartile ranges (IQR), as appropriate. The Shapiro–Wilk test assessed the normality of continuous variables. Normally distributed data were analyzed using unpaired t-tests for group comparisons and paired *t*-tests for within-group comparisons. Non-parametric data were analyzed using the Mann–Whitney and Wilcoxon tests. Categorical data were analyzed using Fisher’s exact and Chi-square tests. Survival and heart failure hospitalization outcomes at 1 year were analyzed, stratified by patient groups (CIED, non-CIED, LTR-A, and LTR-B). Kaplan–Meier survival curves were generated for each group, and comparisons were performed using the log-rank (Mantel-Cox) test to assess statistical significance. Statistical significance was set at *p* = 0.05 (two-tailed). All analyses were conducted using Graphpad Prism 10 (GraphPad Software, San Diego, USA).

## Results

### Baseline clinical characteristics

A total of 204 patients underwent TTVA for severe, symptomatic TR, including 41 (20%) identified as CIED-carriers (Suppl. Figure 1). Baseline characteristics are summarized in Table 1. The median age of CIED-carriers was 79 years and the majority were women (73%) with a median BMI of 26 kg/m (IQR 19.4–33.2). CIED-carriers had a higher perioperative risk (TRI-SCORE: 26% vs. 20%; EuroSCORE II: 7% vs. 5%) and more advanced chronic kidney disease (CKD) (68% with eGFR < 45 ml/min). Most (93%) presented in NYHA-FC ≥ III. Characteristics of LTR-A were seen in 24% of CIED-carriers (10/41), and 44% had torrential TR, compared to 38% in non-CIED-carriers (*p* = 0.592).

**Table 1:** Baseline characteristics

	All patients (*n* = 204)	CIED patients (*n* = 41)	Non-CIED patients (*n* = 163)	*p*-value#
Age (years)*	79 (74–82)	79 (75–83)	79 (74–82)	0.925
Women	77.5% (158/204)	73.17% (11/41)	78.53% (128/163)	0.531
BMI (kg/m^2^)*	25.98 (22.6–30.1)	26.29 (19.4–33.2)	26.17 (18.7–33.6)	0.969
EuroSCORE II (%)	5.67 ± 5.75	6.99 ± 6.34	5.34 ± 5.57	0.036*
TRI-SCORE*	5 (4–6)	6 (5–7)	5 (4–6)	0.120
TRI-SCORE (%)	21.03 ± 16.49	25.56 ± 18.91	19.89 ± 15.68	0.065
*NYHA functional class*				0.026*
II	10.3% (21/204)	7.32% (3/41)	11.04% (18/163)	
III	82.4% (168/204)	75.61% (31/41)	84.05% (137/163)	
IV	7.4% (15/204)	17.07% (7/41)	4.91% (8/163)	
*TR classification*				
Secondary TR	79.9% (163/204)		100% (163/163)	
LTR-A due to RV-lead impingement	4.9% (10/204)	24.39% (10/41)		
LTR-B (incidental)	15.2% (31/204)	75.61% (31/41)		
*TR severity (TTE)*				0.478 0.592
Severe	34.8% (71/204)	26.83% (11/41)	36.81% (60/163)	
Massive	26% (53/204)	29.27% (12/41)	25.15% (41/163)	
Torrential	39.2% (80/204)	43.9% (18/41)	38.03% (62/163)	
*Comorbidities*				
Heart failure with preserved ejection fraction	93.1% (190/204)	90.24% (37/41)	93.87% (153/163)	0.487
NTproBNP (pg/ml)	3250 ± 4468	4066 ± 4654	3035 ± 4408	0.090
Coronary artery disease	37.3% (76/204)	41.46% (17/41)	36.2% (59/163)	0.589
Diabetes mellitus	25.5% (52/204)	26.83% (11/41)	25.15% (41/163)	0.842
Peripheral artery disease	5.9% (12/204)	7.32% (3/41)	5.52% (9/163)	0.711
Chronic obstructive pulmonary disease	18.1% (37/204)	17.07% (7/41)	18.52% (30/162)	> 0.999
Atrial fibrillation	89.7% (183/204)	95.12% (39/41)	88.34% (144/163)	0.260
Prior Stroke	17.6% (36/204)	7.32% (3/41)	20.25% (33/163)	0.066
Advanced CKD (eGFR (FAS) < 45 ml/min)	50% (102/204)	68.29% (28/41)	45.4% (74/163)	0.014*
Dialysis	5.4 (11/204)	4.88% (2/41)	5.52% (9/163)	> 0.999
Creatinine (mg/ml)	1.36 ± 0.73	1.62 ± 1.02	1.3 ± 0.62	0.038*
eGFR (FAS) ml/min	47.73 ± 19.74	40.92 ± 17.02	49.48 ± 20.04	0.012*
*Transthoracic echocardiographic variables*				
LVEF (%)	55.17 ± 9.44	51.78 ± 11.09	55.96 ± 8.82	0.049*
RV basal diameter (mm)	47.23 ± 8.65	46.88 ± 10.85	47.32 ± 8.03	0.560
TAPSE (mm)	18.10 ± 5.02	17.74 ± 4.55	18.18 ± 5.135	0.820
TR effective regurgitation orifice area (cm^2^)	0.76 ± 0.39	0.89 ± 0.79	0.73 ± 0.32	0.191
TR vena contracta (mm)	14.48 ± 5.37	15.41 ± 5.64	14.24 ± 5.29	0.251
TR regurgitation volume (ml)	58.51 ± 26.31	61.31 ± 32.85	57.83 ± 24.52	0.964
Systolic pulmonary artery pressure (mmHg)	37.51 ± 13.53	35.95 ± 12.83	37.88 ± 13.71	0.435
Inferior vena cava diameter (mm)	24.70 ± 6.92	25.55 ± 7.29	24.5 ± 6.84	0.207

Values are presented in Percent (%), Mean ± Standard deviation (SD), or as Median* ± interquartile range (IQR). Significant p-values are emphasized in bold

*BMI* Body mass index, *NYHA* New York Heart Association, *TR* Tricuspid regurgitation, *CKD* Chronic kidney disease, *GFR* Glomerular filtration rate, *TTE* Transthoracic echocardiography, *LVEF* Left ventricular ejection fraction, *RV* Right ventricle, *RA* Right atrium, # comparison between CIED and non-CIED patients

### Procedural outcome and safety endpoints

Tables 2 and 3 outline procedural characteristics and safety endpoints. TVARC intraprocedural success was similar between CIED carriers (68%) and controls (70%). Echocardiographic variables according to intraprocedural success in CIED and non-CIED patients are provided in Supl. Table 2. Procedure times, radiation doses, and hospital stays were comparable across groups. Despite similar baseline TR severity (*p* = 0.472), the subgroup of LTR-A patients had significantly worse TR reduction at discharge compared to LTR-B patients, with median reductions of 1 TR grade versus 2 TR grades, respectively (*p* = 0.022) (Table 4).

**Table 2:** Procedural characteristics

	All patients (*n* = 204)	CIED patients (*n* = 41)	Non-CIED patients (*n* = 163)	*p*-value#
Length of hospitalisation (d)	9.67 ± 9.33	10.38 ± 8.3	9.49 ± 9.58	0.800
Procedure time (min)	197.9 ± 57.21	190.7 ± 47.83	199.7 ± 59.31	0.667
Radiation dose (cGy cm^2^)	11,453 ± 9967	12,265 ± 9496	11,261 ± 10,100	0.369
Contrast medium volume (ml)	109.6 ± 57.82	89.3 ± 42.48	112.5 ± 57.9	0.039*
Annulus perimeter (mm) (measured 4 mm from annulus)	111.97 ± 16.07	109.8 ± 14.79	112.7 ± 16.38	0.445
Anteroseptal Annulus diameter (mm)	42.69 ± 6.04	43.43 ± 4.63	42.5 ± 6.35	0.390
*Implant size*				0.857
C	1.5% (3/196)	2.5% (1/40)	1.28% (2/156)	
D	8.2% (16/196)	7.5% (3/40)	8.33% (13/156)	
E	24% (47/196)	20% (8/40)	25% (39/156)	
F	66.3% (130/196)	70% (28/40)	65.38% (102/156)	
*TR severity post-band (TEE)*				0.203
No/mild	42% (84/200)	31.71% (13/41)	44.65% (71/159)	
Moderate	31% (62/200)	39.02% (16/41)	28.93% (46/159)	
Severe	18.5% (37/200)	24.39% (10/41)	16.98% (27/159)	
Massive	6.5% (13/200)	2.44% (1/41)	7.55% (12/159)	
Torrential	2% (4/200)	2.44% (1/41)	1.89% (3/159)	
*Grade of TR reduction post-band (TEE)*				0.994
0	3.5% (7/200)	2.44% (1/41)	3.77% (6/159)	
1	12% (24/200)	12.2% (5/41)	11.95% (19/159)	
2	46% (92/200)	46.34% (19/41)	45.91% (73/159)	
3	28% (56/200)	29.27% (12/41)	27.67% (44/159)	
4	10.5% (21/200)	9.76% (4/41)	10.69% (17/159)	
TVARC intraprocedural success	69.6% (142/204)	68.29% (28/41)	69.94% (115/163)	0.851
*TR severity at discharge (TTE)*				0.919
No/mild	31.1% (62/199)	25% (10/40)	32.7% (52/159)	
Moderate	32.2% (64/199)	32.5% (13/40)	32.08% (51/159)	
Severe	24.6% (49/199)	27.5% (11/40)	23.9% (38/159)	
Massive	10.6% (21/199)	12.5% (5/40)	10.06% (16/159)	
Torrential	1.5% (3/199)	2.5% (1/40)	1.26% (2/159)	

Values are presented in Percent % or as Mean ± SD. Standard deviation. Significant p-values are emphasized in bold

*BL* Baseline, *TEE* Transesophageal echocardiography, *RCA* Right coronary artery, *RBBB* Right bundle branch block, *SSS* Sick.Sinus-Syndrome, *PEA* Pulseless electrical activity # comparison between CIED and non-CIED patients

**Table 3:** Safety endpoints

Device- and procedure-related complications	All patients (*n* = 204)	CIED patients (*n* = 41)	Non-CIED patients (*n* = 163)	*p*-value#
Device detachment	1% (2/204)	0% (0/41)	1.23% (2/163)	> 0.999
RCA stenting	4.9% (10/204)	2.5% (1/40)	5.59% (9/161)	0.690
Cardiac injury requiring	2.5% (5/204)	0% (0/41)	3.07% (5/163)	0.253
Pericardiocentesis	2% (4/204)	0% (0/41)	2.45% (4/163)	0.308
Emergency cardiac surgery	0.5% (1/204)	0% (0/41)	0.61% (1/163)	> 0.999
*30 day events*				
Conduction disturbances	12.7% (26/204)	14.63% (6/41)	12.27% (20/163)	> 0.999
RBBB	0.5% (1/204)	0% (0/41)	0.61% (1/163)	
Bradycardia/SSS	4.4% (9/204)	2.44% (1/41)	4.91% (8/163)	
Atrial fibrillation	2.9% (6/204)	2.44% (1/41)	3.07% (5/163)	
Non-fatal ventricular arrythmia	2% (4/204)	4.88% (2/41)	1.23% (2/163)	
New CIED	1.5% (3/204)	2.44% (1/41)	1.84% (3/163)	
Fatal arrythmias	1% (2/204)	2.44% (1/41)	0.61% (1/163)	
Complications involving CIEDs		12.2% (5/41)		
Lead dislodgement		2.44% (1/41)		
Stroke	1.5% (3/204)	0% (0/41)	1.84% (3/163)	> 0.999
TVARC Bleeding (Type 5)	0% (0/204)	0% (0/41)	0% (0/163)	
TVARC acute kidney injury (Stage 4)	5.4% (11/204)	4.88% (2/41)	5.52% (9/163)	> 0.999
In-hospital mortality	2.5% (5/204)	2.44% (1/41)	2.45% (4/163)	> 0.999

Values are presented in Percent % or as Mean ± SD. Standard deviation;

*BL* Baseline, *TEE* Transesophageal echocardiography, *RCA* Right coronary artery, *RBBB* Right bundle branch block, *SSS* Sick.Sinus-Syndrome, *PEA* Pulseless electrical activity # comparison between CIED and non-CIED patients

**Table 4:** CIED-related subgroup analysis

Echocardiographic variables at baseline (BL)	LTR-A (n = 10)	LTR-B (n = 31)	*p*-value
LVEF % BL	50.7 ± 9.17	52.1 ± 11.76	0.421
*TR severity at BL*			0.472
Severe	40% (4/10)	22.58% (7/31)	
Massive	30% (3/10)	29.03% (9/31)	
Torrential	30% (3/10)	48.38% (15/31)	
Δ TR severity discharge vs. BL (TTE)	- 1.4 ± 0.7	- 2.03 ± 0.85	0.022*
Echocardiographic variables at 30-day follow-up	LTR-A (n = 8)	LTR-B (n = 24)	
*TR severity FU*			**0.041**
No/mild	12.5% (1/8)	25% (6/24)	
Moderate	25% (2/8)	37.5% (9/24)	
Severe	25% (2/8)	37.5% (9/24)	
Massive	12.5% (1/8)	0% (0/24)	
Torrential	25% (2/8)	0% (0/24)	
Δ TR severity FU vs. discharge (TTE)*	1 (1–0)	0 (0–1)	0.075
*NYHA functional class at FU*			0.058
I	25% (2/8)	4.17% (1/24)	
II	25% (2/8)	62.5% (15/24)	
III	37.5% (3/8)	33.33% (8/24(	
IV	12.5% (1/8)	0% (0/24)	
Need for heart failure hospitalization at 1 year (*n* = 35)	33.33% (3/9)	3.85% (1/26)	0.044*
Loop diuretics at FU (mg)	98.75 ± 94.18	35.91 ± 31.42	0.047*
eGFR (FAS) at FU ml/min	36.13 ± 5.36	44.71 ± 17.4	0.186

Values are presented in Percent % or as Mean ± SD. Standard deviation or Median* ± interquartile range (IQR). Significant p-values are emphasized in bold

*BL* Baseline, *FU* Follow-up (mean 73 ± 41 days), *GFR* Glomerular filtration rate, *LVEF* Left ventricular ejection fraction, *NYHA* New York Heart Association, *RV* Right ventricle, *RA* Right atrium, *TTE* Transthoracic echocardiography, *TR* Tricuspid regurgitation, Δ Delta (difference between two time points)

In-hospital mortality was below 3%. Fatal ventricular arrhythmias occurred in one CIED and one non-CIED-carrier on the third and fourth postinterventional day respectively, both with pre-existing coronary heart disease. The CIED carriers` Holter monitor showed multiple ventricular tachycardia episodes, one near the time of death. The patient had a known history of chronic left ventricular heart failure and no direct relation to the procedure was found.

Overall, conduction disturbances occurred in 13%, most of them being clinically insignificant and reversible (Table 3). Three non-CIED carriers (1.8%) required new CIED implantation due to acute, persistent third-degree atrioventricular (AV)-Block. One patient experienced pacemaker-induced tachycardia (PMT) due to increased lead traction during atrial RV lead passage, resolved by magnetic application. Anatomical variations (laterally placed inferior vena cave (IVC) orifice and Cor triatriatum) caused periprocedural septal displacement of RV leads in two patients without conduction disturbances or significant changes in CIED parameters. One CIED-carrier experienced complete RV lead dislodgment, leading to an imminent third-degree AV block, necessitating emergency leadless RV pacemaker implantation, and was discharged 14 days later to a geriatric rehabilitation facility with TR reduction from IV to II.

### Periprocedural CIED interrogations

Among the 41 CIED-carriers, 24 completed standardized pre- and postprocedural CIED interrogations at the participating hospitals (Table 5). A significant reduction in remaining operational lifespan (ROL) of 3 months was noted post-TTVA (*p* = 0.026), accounted for by the elapsed time between CIED interrogations. RA lead impedance was significantly reduced (*p* = 0.018), but not clinically relevant in any of the examined CIED carriers according to TVARC (change in pacing lead impedance of > 200 Ω). There was a significant difference between pre- and post-procedural RV lead sensing (11.7 mV vs. 10. 3 mV; *p* = 0.031), though RV pacing parameters remained stable.

**Table 5:** Periprocedural CIED interrogations

	Pre-intervention (*n* = 24)	Post-intervention (*n* = 24)	*p*-value
*CIED interrogation parameters*			
Remaining operational lifespan (months)	62.51 ± 39.23	59.07 ± 38.87	**0.026**
Δ Elapsed time between CIED interrogations and ROL differences pre- and post-Intervention (months)		1.95 ± 7.27	0.389
*RA-Lead* (*n* = 10)			
Atrial pacing (%)	13.41 ± 27.41	12.41 ± 19.93	0.656
Sensing (mV)	1.54 ± 0.97	1.27 ± 0.6	0.147
Impedance (Ohm)	471 ± 103.7	424.1 ± 89.57	0.018*
Output (pulse strength) (V)	2.23 ± 0.84	2.4 ± 0.75	0.360
Output (pulse duration) (ms)	0.89 ± 1.18	0.52 ± 0.22	0.500
*RV-Lead* (*n* = 24)			
Ventricular pacing (%)	69.19 ± 38.09	71.11 ± 37.44	0.629
Sensing (mV)	11.68 ± 4.07	10.26 ± 3.29	0.031*
Capture threshold (V)	0.94 ± 0.4	0.89 ± 0.26	0.915
Impulse duration of capture threshold (ms)	0.51 ± 0.28	0.51 ± 0.28	> 0.999
Impedance (Ohm)	503.1 ± 204.9	505.8 ± 229.6	0.791
Output (pulse strength)	1.99 ± 0.59	1.93 ± 0.58	0.371
Output (pulse duration) (ms)	0.5 ± 0.28	0.5 ± 0.28	> 0.999
*LV-Lead* (*n* = 3)			
Biventricular pacing (%)	98.7 ± 0.48	98.63 ± 0.48	0.391
Capture threshold (V)	0.67 ± 0.14	0.83 ± 0.29	0.500
Impulse duration of capture threshold (ms)	1 ± 0.5	1 ± 0.5	> 0.999
Impedance (Ohm)	547.3 ± 184.7	474.7 ± 149.1	0.097
Output (pulse strength)	1.5 ± 0.43	1.67 ± 0.29	0.500
Output (pulse duration) (ms)	1 ± 0.5	1 ± 0.5	> 0.999

Values are presented in Percent % or as Mean ± SD. Standard deviation. Significant p-values are emphasized in bold

*ICD* Implantable cardioverter defibrillator, *CRT* Cardiac resynchronization therapy, *RV* Right ventricle, *RA* Right atrium, *LV* Left ventricle

### Echocardiographic outcome at follow-up

At 30-day follow-up (FU), TTE data from 157 patients, including 32 CIED carriers, showed significant TR severity reduction post-TTVA (*p* < 0.0001) (Supl. Table 3). Residual TR ≤ II was seen in 56% of CIED carriers and 68% of non-CIED carriers (*p* = 0.756) (Table 6). TR was significantly worse in the small subgroup of LTR-A patients compared to LTR-B at FU (*p* = 0.041) (Table 4). CIED carriers with LTR-A showed a trend for progressive TR worsening from discharge to follow-up, while LTR-B patients showed marginal improvements (*p* = 0.075).

**Table 6:** Echocardiographic variables at 30-day follow-up

	All patients (*n* = 157)	CIED patients (*n* = 32)	Non-CIED patients (*n* = 125)	*p*-value#
LVEF (%)	56.11 ± 8.89	50.58 ± 11.83	57.52 ± 7.4	< 0.001***
RV basal diameter (mm)	41.67 ± 7.34	41.75 ± 7.59	41.5 ± 7.63	0.812
RV FAC (%)	37.35 ± 10.9	37.45 ± 9.01	37.33 ± 11.38	0.959
RA area (cm^2^)	29.43 ± 9.56	29.32 ± 7.48	29.45 ± 10.03	0.665
TR EROA (cm^2^)	0.41 ± 0.92	0.34 ± 0.24	0.42 ± 1.02	0.085
Δ TR EROA FU vs BL (cm^2^)	-0.38 ± 0.94	-0.51 ± 0.4	-0.34 ± 1.03	0.698
TR vena contracta (mm)	6.85 ± 4.4	6.73 ± 3.93	6.88 ± 4.53	0.800
Δ TR vena contracta FU vs BL (mm)	-7.72 ± 5.26	-8.36 ± 4.6	-7.57 ± 5.42	0.467
TR regurgitation volume (ml)	25.69 ± 19.6	26.46 ± 16.45	25.5 ± 20.38	0.434
Δ TR regurgitation volume FU vs BL (ml)	-31.23 ± 25.24	-28.33 ± 22.33	-31.01 ± 24.37	0.624
sysPAP (mmHg)	44.85 ± 15.68	40.94 ± 15.54	45.81 ± 15.64	0.119
IVCd (mm)	20.28 ± 5.77	20.46 ± 5.18	20.23 ± 5.93	0.850
Δ IVCd FU vs BL (mm)	-4.1 ± 6.69	-3.08 ± 9.16	-4.35 ± 5.98	0.407
*TR severity at follow-up (TTE)*				0.756
No/mild	26.75% (42/157)	21.88% (7/32)	28% (35/125)	
Moderate	38.85% (61/157)	34.38% (11/32)	40% (50/125)	
Severe	25.48% (40/157)	34.38% (11/32)	23.2% (29/125)	
Massive	6.3% (8/157)	3.13% (1/32)	5.6% (7/125)	
Torrential	3.82% (6/157)	6.25% (2/32)	3.2% (4/125)	
*TR severity ≤ II*	65.61% (103/157)	56.25% (18/32)	68% (85/125)	0.219

Values are presented in Percent (%), Mean ± Standard deviation (SD), or Median* ± interquartile range (IQR). Significant p-values are emphasized in bold

*RV* Right ventricle, *RA* Right atrium, *BL* Baseline, *FU* Follow-up (mean 73 ± 41 days), Δ Delta (difference between two time points) # comparison between CIED and non-CIED patients

### Clinical outcomes at follow up

**Fig. 1:**
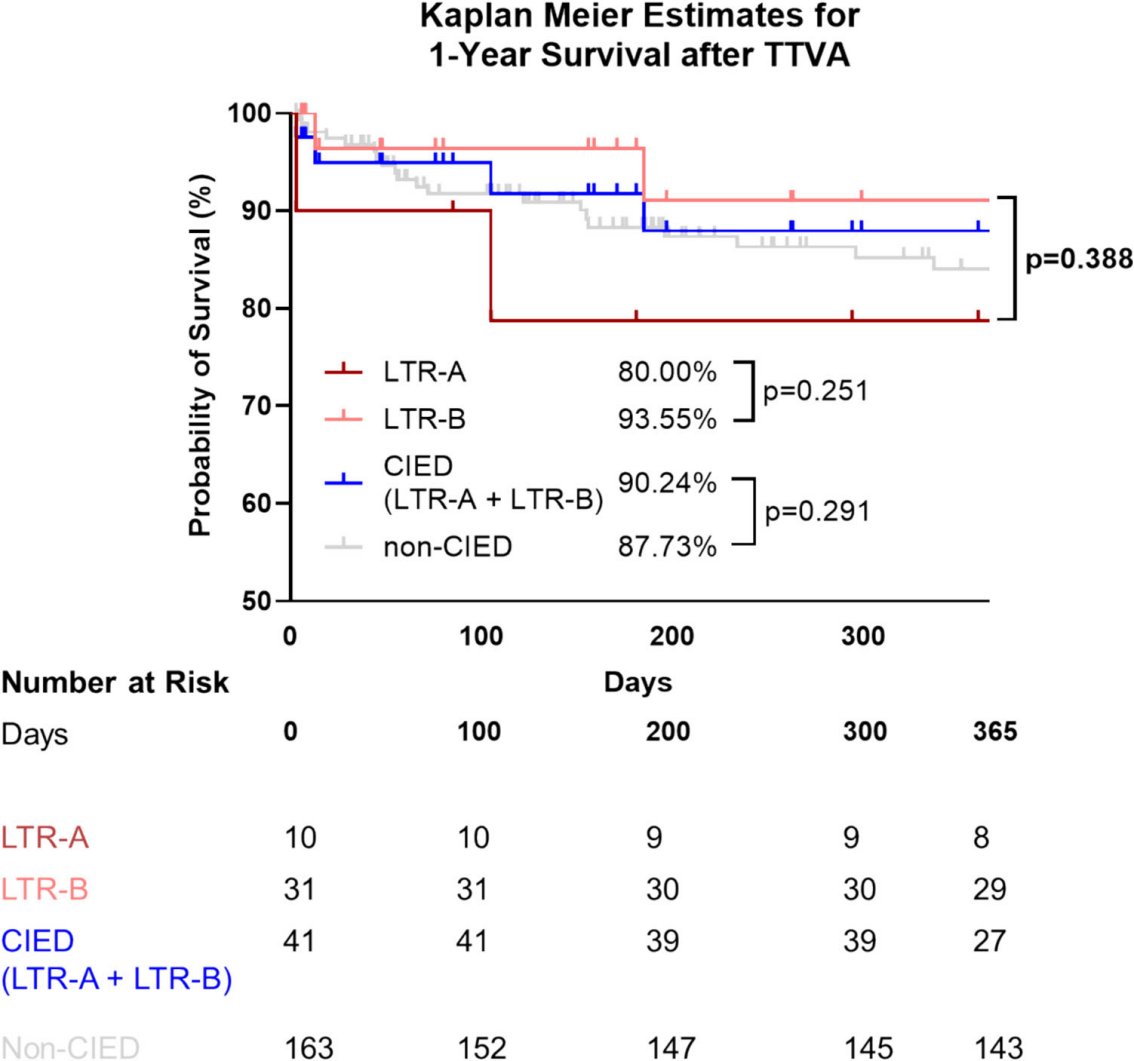
Overall survival of CIED- and non-CIED-carriers after TTVA. One-year landmark analysis of overall survival in individuals treated with TTVA. There was no significant difference between CIED- and non-CIED-carriers (Log-rank (Mantel-Cox) test; *p* = 0.291). The *p*-value for trend, representing the overall comparison of survival curves (Log-rank (Mantel-Cox) test), is highlighted in bold

During a 30-day follow-up period, all-cause mortality was 5% in CIED carriers and 3% in controls (Table 7). Most patients showed improvement in NYHA-FC and a decrease in NTproBNP levels, although CIED patients had higher NTproBNP levels at FU (*p* = 0.012). Heart failure re-hospitalization rates were similar between CIED (11%) and non-CIED (13%) carriers (*p* > 0.999) at 1 year. LTR-A patients tended to have more severe dyspnea at FU compared to LTR-B candidates (50% LTR-A vs. 33.33% LTR-B; p = 0.058) (Table 7). There were more heart failure hospitalizations in LTR-A patients (33.3% vs. 3.85%; p = 0.012) (Supl. Figure 2), along with higher daily loop diuretic intake (99 mg vs. 36 mg furosemide equivalent dose; p = 0.047). One-year landmark analysis of overall survival was comparable between CIED (10%) and non-CIED (12%) patients (p = 0.291), though LTR-A patients showed a trend towards decreased survival compared to LTR-B (p = 0.251) (Fig. 1).

**Table 7:** Clinical outcomes at 30-day follow-up

	All patients (*n* = 157)	CIED patients (*n* = 32)	Non-CIED patients (*n* = 125)	*p*-value#
*Circulating biomarkers and end-organ function*				
NTproBNP at FU (pg/ml)	2757 ± 3923	4105 ± 6702	2346 ± 2456	0.012*
Δ NTproBNP FU vs. BL (pg/ml)	-301 ± 3076	-380.4 ± 3667	-275.4 ± 2883	0.949
Creatinine at FU (mg/ml)	1.41 ± 0.98	1.56 ± 1.4	1.37 ± 0.84	0.302
Δ Creatinine FU vs. BL (mg/ml)	0.08 ± 0.61	-0.06 ± 0.78	0.12 ± 0.55	0.504
eGFR (FAS) at FU ml/min	45.73 ± 16.99	42.34 ± 15.45	46.65 ± 17.34	0.228
Δ eGFR (FAS) FU vs. BL ml/min	-2.22 ± 9.15	-0.76 ± 8.15	-2.63 ± 9.41	0.331
*Clinical outcome:*				
NYHA functional class at FU				0.617
I	7.6% (12/157)	9.38% (3/32)	7.2% (9/125)	
II	60.5% (95/157)	53.13% (17/32)	62.4% (78/125)	
III	30.6% (48/157)	34.38% (11/32)	29.6% (37/125)	
IV	1.3% (2/157)	3.13% (1/32)	0.8% (1/125)	
NYHA functional class ≤ II at FU	68.15% (107/157)	62.51% (20/32)	69.6% (87/125)	0.524
Δ NYHA functional class FU vs. BL				0.722
-2	10.8% (17/157)	15.63% (5/32)	9.6% (12/125)	
-1	53.5% (84/157)	50% (16/32)	54.4% (68/125)	
0	33.8% (53/157)	31.25% (10/32)	34.4% (43/125)	
1	1.9% (3/157)	3.13% (1/32)	1.6% (2/125)	
Need for heart failure hospitalization at 1 year	13.07% (23/176)	11.43% (4/35)	13.45% (19/141)	> 0.999
All-cause mortality at FU	3.43% (7/204)	4.88% (2/41)	3.07% (5/163)	0.630
*Need of diuretics*				
Loop diuretics (Furosemid equivalent dose mg) at BL	69.02 ± 105.09	73.1 ± 89.4	68 ± 108.9	0.473
Loop diuretics (Furosemid equivalent dose mg) at FU	66.57 ± 110.56	76.77 ± 146.8	64.04 ± 100.2	0.742
Δ Loop diuretics FU vs, BL (mg)	3.03 ± 56.34	11.1 ± 79.96	1 ± 48.93	0.518
Sequential nephron blockade	14.19% (22/155)	22.58% (7/31)	12.10% (15/124)	0.153
Mineralocorticoid receptor antagonist (MRA)	52.9% (82/155)	58.06% (18/31)	51.61% (64/124)	0.552

Values are presented in % or as mean/median* ± SD. Standard deviation. Significant p-values are emphasized in bold

*BL* Baseline, *FU* Follow-up, Δ Delta (difference between two time points) # comparison between CIED and non-CIED patients

## Discussion

In this retrospective real-world analysis, we evaluated the feasibility, efficacy, and safety of TTVA in patients with CIEDs, marking the largest study of this select cohort to date. The study highlighted three key findings: (1) TTVA is a safe and effective treatment option for secondary TR in CIED carriers, (2) CIED functionality is largely preserved following TTVA, and (3) LTR-A patients present challenges for TTVA-based TR repair.

In previous TTVA trials, CIED carriers were underrepresented, with only 24 CIED-carriers included in all European and US feasibility studies. Specifically, CIED-carriers comprised 13.3% (n = 4/30) in TRI-REPAIR, 29.7% (n = 11/37) in TR EFS, and 14.8% (n = 9/61) in the post-approval TRI-Band study [11, 21, 23]. While the proportion of CIED-related TR in these trials was consistent with the previously reported incidence of 10–15%, the overall number of CIED carriers treated with TTVA remained low, making post hoc subgroup analyses for this population challenging. For the first time, our large real-world cohort enabled a retrospective analysis of the safety and efficacy of TTVA in CIED patients.

Importantly, our results showed similar intraprocedural success after TTVA in CIED carriers (68.3%) and non-CIED carriers (69.9%), with comparable safety endpoints, procedure time and hospitalization duration.

Compared to previous TTVA trials, a smaller proportion of our CIED carriers achieved moderate or less TR at 30-day follow-up (56% vs. 63–69% in TRI-REPAIR and TRI-Band), likely due to the higher prevalence of advanced baseline TR (73% with massive or torrential TR vs. 52–69%) [21, 23]. Stratification by intraprocedural success revealed that smaller annulus size and less severe TR at baseline were associated with successful periprocedural outcomes in this cohort, regardless of CIED presence (Supl. Table 2). Notably, the above mentioned studies did not specifically compare procedural outcomes between CIED and non-CIED carriers, nor was the discrimination of LTR-A and LTR-B etiology considered. While both CIED- and non-CIED carriers showed similar TTVA outcomes, with 85% achieving ≥ 2-grade TR reduction (p = 0.994), non-CIED carriers had higher rates of moderate or mild residual TR at 30-day follow-up (68% vs. 56%, p = 0.756). This difference was mainly driven by LTR-A patients in the CIED group, who showed less TR reduction at discharge (p = 0.022) and worse TR and dyspnea at 30-day follow-up, requiring more diuretics and heart failure rehospitalizations. Excluding LTR-A cases, similar rates of moderate or less TR were seen in both groups (62.5% LTR-B vs. 68% non-CIED carriers, p = 0.716). One-year survival was comparable (90% for CIED vs. 88% for non-CIED, p = 0.291), but LTR-A patients trended towards higher mortality (20% vs. 6% for LTR-B, p = 0.251) (Fig. 1). These subgroup analyses, while only hypothesis-generating due to the retrospective design and small sample size, underscore potential inferior device performance in LTR-A patients. Currently, T-TEER remains the dominant catheter-based repair for TR, even in complex anatomies. However, LTR-A patients have limited therapeutic options with high screening failure rates for interventional TV repair due to the interactions of leaflets with CIED leads [6, 10, 12]. In our cohort, almost 50% of all CIED leads traversed the TV centrally, likely making these patients unsuitable for T-TEER due to potential lead-device interaction, leading to their screening for TTVA instead. While T-TEER trials reported better clinical success (77–86% vs. 56% in our CIED TTVA cohort), direct comparisons between TTVA and T-TEER for CIED patients, especially with LTR-A features, are unavailable [20, 24, 27]. Notably, TTVA achieved similar procedural success, with 91% of CIED patients showing at least one grade TR reduction, comparable to T-TEER results in CIED carriers (85–92%) at 30 days [2, 19]. Screening for dedicated transcatheter tricuspid valve replacement (TTVR) may benefit LTR-A patients, as shown by the TRISCEND study, where TTVR led to sustained TR reduction in all 5 LTR-A patients to moderate or less at 1-year FU [16]. More research is needed on CIED patient selection, especially since TTVI has shown better outcomes and symptomatic improvement than optimal medical therapy alone [24].

CIED patients in this study had a significantly higher perioperative risk than non-CIED controls, mainly due to advanced kidney disease (elevated EuroScore II and TRI-SCORE). The cumulative incidence of procedural complications seemed numerically lower in CIED patients compared to non-CIED patients (10% (4/41) vs. 15% (24/163); p = 0.611), possibly due to the later inclusion of CIED patients and lower sample size, with cases increasing significantly from 2013 to 2023 (Supl. Figure 3A). Overall complication rates remained unchanged over time (Supl. Figure 3B). In 2023, complication rates were equal among CIED and non-CIED patients (Supl. Figure 3C,D). All-cause 30-day mortality in our cohort was higher than in other registries (4.88% vs. 0–1.6%), likely due to the greater morbidity and symptom severity in our patients (17–20). Nevertheless, isolated CIED complications were rare. Only one of the 41 CIED carriers experienced lead dislodgement, leading to third-degree AV block, requiring emergency implantation of a leadless pacemaker. In this particular case, the presence of two RV leads complicated the placement of the device guide catheter in the RA. In contrast, 3 non-CIED patients (1.84%) required CIED implantation post-TTVA due to bradycardia or AV block, possibly from anchor-induced RCA stenosis, that could not be resolved with anchor removal or stenting. This aligns with previously reported CIED rates after TTVA (0–3.3%) [11, 21, 23].

We are pleased to report minimal changes in pacing parameters during periprocedural CIED interrogations, none of which had clinical significance, according to TVARC criteria [13]. At 30-day follow-up, no critical CIED dysfunction requiring unplanned intervention occurred. We, therefore, conclude that our observed CIED changes do to not play a major role for daily clinical practice. These minor changes are likely due to mechanical interference between the device sheath and CIED leads during valve repair, as the TTVA approach involves passing the leads laterally after entering the RA from the IVC. In most cases, the device is placed on the TV annulus from anterior to the coronary sinus region, avoiding critical lead interaction. However, variations in lead position and mechanical properties make pre-interventional planning crucial. This includes 3D-TEE, CT screening, and intraoperative echocardiographic guidance to assess lead position and mobility, reducing the risk of lead interference during valve interventions.

Overall, TTVA remains an effective and safe option for CIED carriers with elevated preoperative risk.

### Study limitations

The retrospective nature of our study, limited acquisition of all parameters, and cohort size may constrain statistical power to detect subtle effects in our study. Moreover, standardized serial CIED interrogations were only available for 24 out of 41 CIED carriers, further diminishing the statistical power in an already moderately small cohort. The other 17 CIED carriers received CIED interrogations at the referring hospitals or outpatient units and had to be excluded from the final analysis due to high heterogeneity in the completeness of CIED interrogation data. Our clinical and echocardiographic FU aligns with previous studies on TR interventions in CIED cohorts [4, 25]. However, the results are derived from centers with substantial case volumes and may not be generalized broadly, as TTVA is a technically complex procedure.

## Conclusion

In this real-world study, TTVA was overall equally successful in CIED- and non-CIED-carriers. In the small subgroup of LTR-A patients, procedural success was less frequent compared to LTR-B. While overall device complications were low, one case of lead dislodgement occurred, requiring emergency pacemaker-implantation. Post-interventional CIED interrogations showed no critical technical issues. These findings affirm the safety and efficacy of TTVA in CIED-carriers, even in, however, less so, in LTR-A patients, highlighting the necessity for thorough pre-procedural evaluation and vigilant post-operative monitoring.

## Supplementary Information

Below is the link to the electronic supplementary material.

Supplementary file1 (DOCX 392 KB)

## Data Availability

The data that support the findings of this study are not openly available due to reasons of sensitivity and are available from the corresponding author upon reasonable request. Data are located in controlled access data storage at University Hospital Cologne.

## Abbreviations

AKI: Acute kidney injury
CIED: Cardiac implantable electronic device
CRT: Cardiac resynchronization therapy
eGFR: Estimated glomerular filtration rate
LTR-A: Lead-associated tricuspid regurgitation, type A (causative)
LTR-B: Lead-associated tricuspid regurgitation, type B (unrelated)
NYHA-FC: New York heart association functional class
PVR: Pulmonary vascular resistance
RA: Right atrium
RV: Right ventricle
TEE: Transesophageal echocardiography
TEER: Transcatheter edge-to-edge repair
TR: Tricuspid regurgitation
TTE: Transthoracic echocardiography
TTVA: Transcatheter tricuspid valve annuloplasty
TVARC: Tricuspid valve academic research consortium
