# Supplementary material for: Cardiac implantable electronic device carriers undergoing transcatheter tricuspid valve annuloplasty: real-world insights
Source: Clin Res Cardiol. 2025 Mar 10;114(7):878–91. doi: 10.1007/s00392-025-02616-5 (PMC12202581; doi:10.1007/s00392-025-02616-5)
Supplement: Supplementary file 1 — Supplementary file1 (DOCX 392 KB) [file 392_2025_2616_MOESM1_ESM.docx]

**SUPPLEMENTAL MATERIAL**

**Supplemental Figures and Figure Legends**


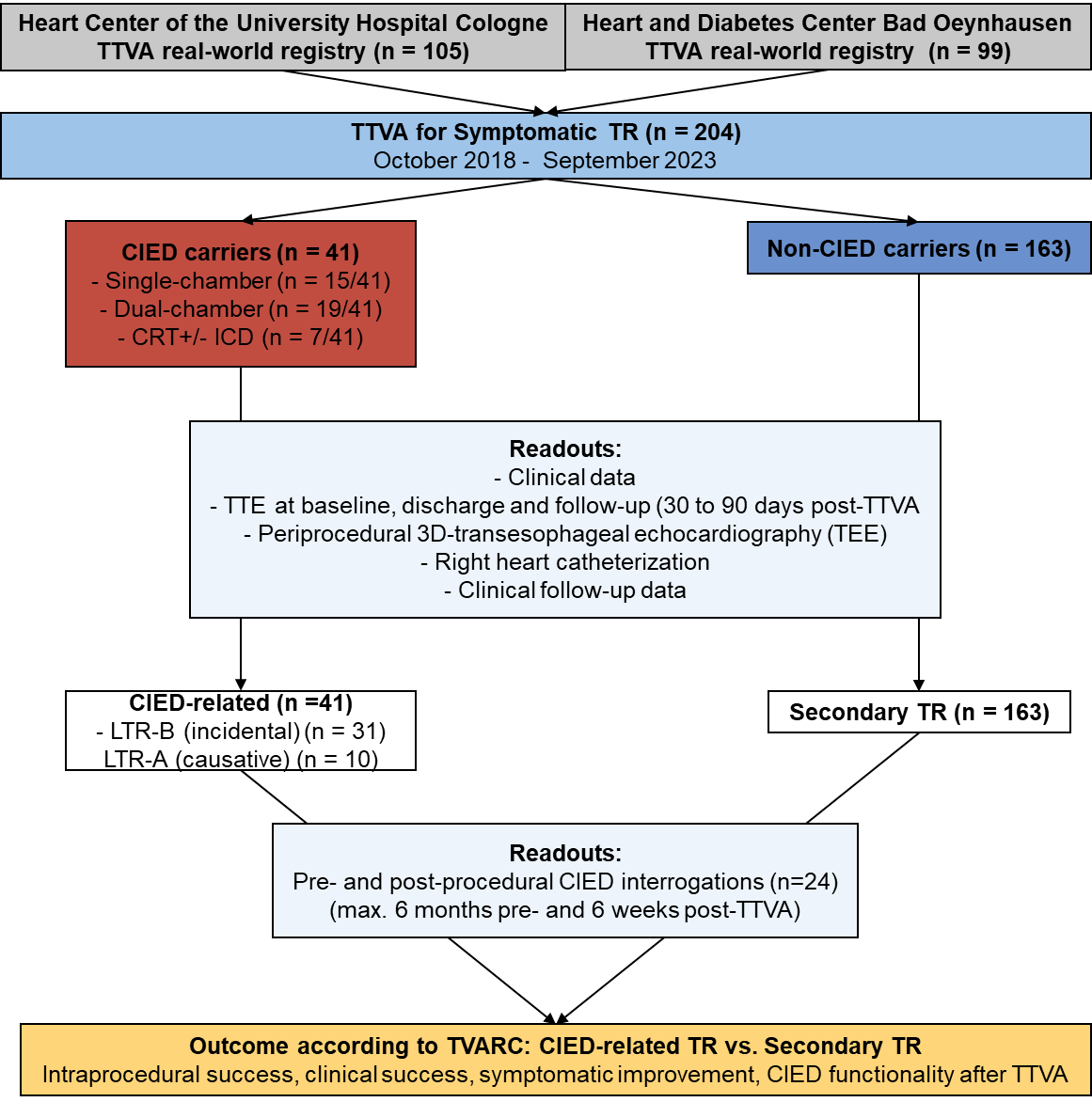


**Supplemental Figure 1: Study Flow chart**

Depiction of patient numbers available for analysis and diagnostic work-up.

CRT = Cardiac Resynchronization Therapy; ICD = Implantable Cardioverter-Defibrillator; LTR-A = Lead-associated Tricuspid Regurgitation, type A; LTR-B = Lead-associated Tricuspid Tegurgitation, type B; TR = Tricuspid Regurgitation; TTVA = Transcatheter Tricuspid Valve Annuloplasty


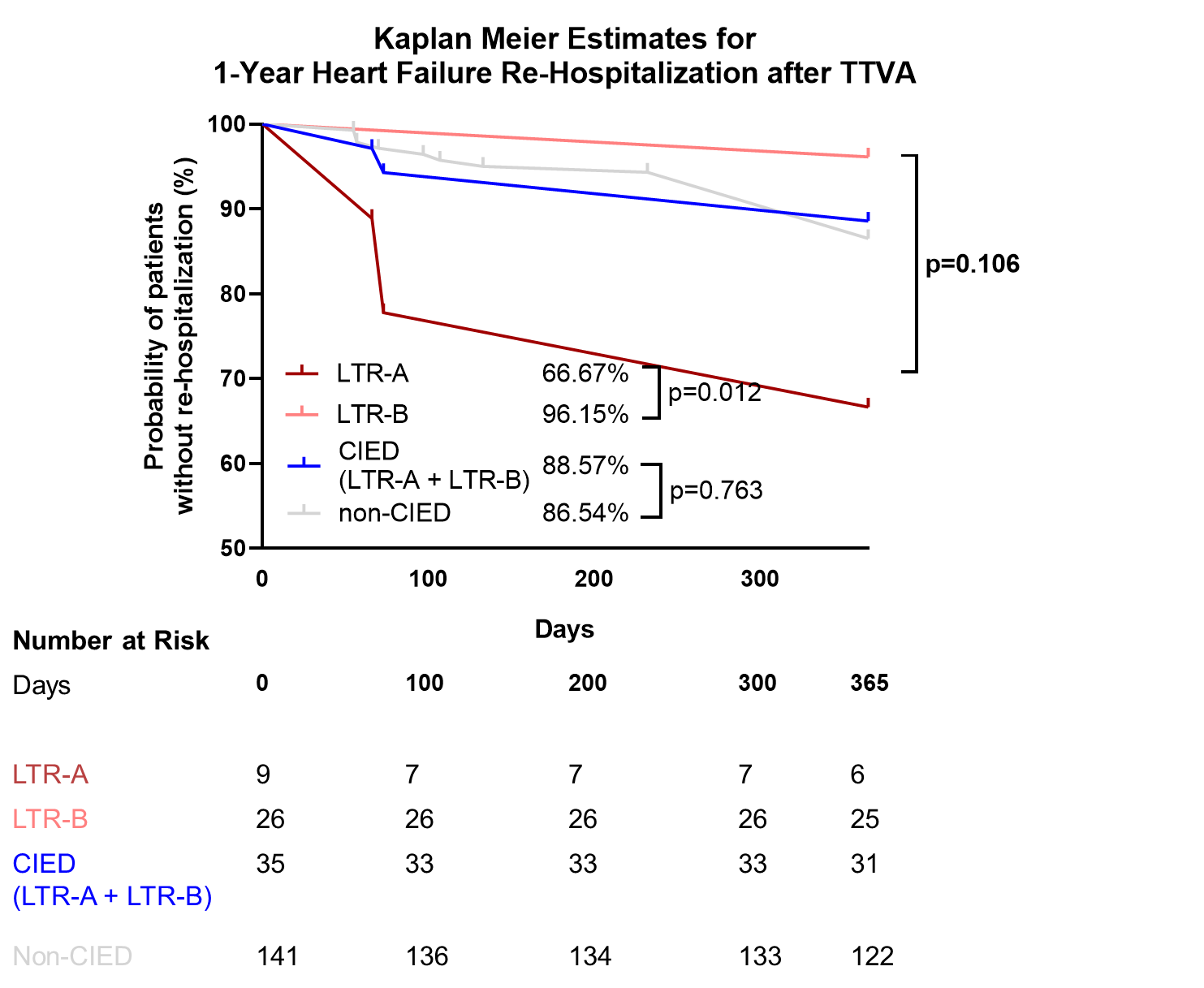


**Supplemental Figure 2: Heart failure re-hospitalization of CIED- and non-CIED -patients following TTVA**

One-year landmark analysis of heart failure re-hospitalization in individuals treated with TTVA. LTR-A patients were significantly more hospitalized than LTR-B patients (Log-rank (Mantel-Cox) test; p=0.012). The p-value for trend, representing the overall comparison of re-hospitalization curves (Log-rank (Mantel-Cox) test), is highlighted in bold.


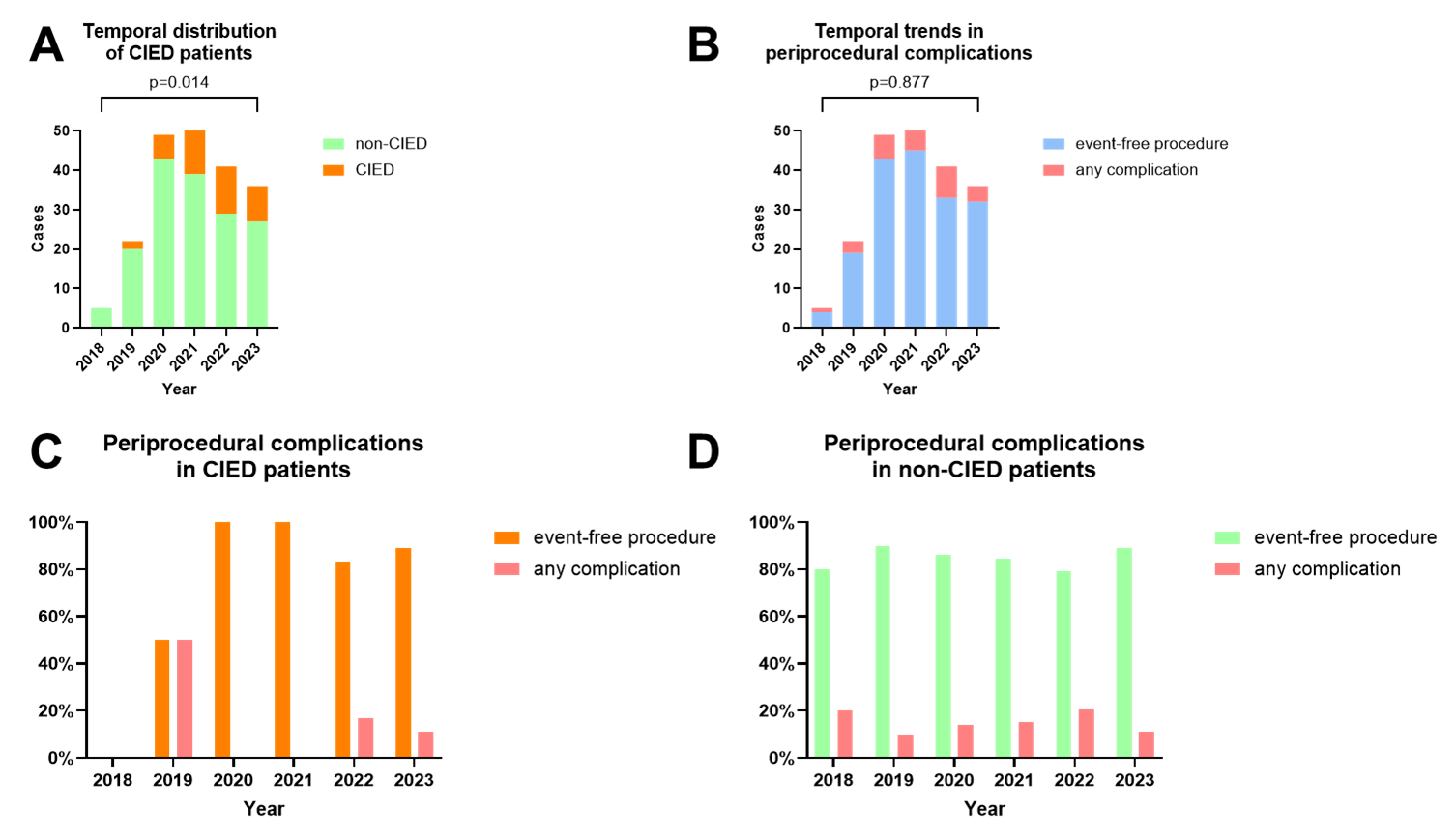


**Supplemental Figure 3: Temporal trends in TTVA treatment and periprocedural complications of CIED and non-CIED patients between 2018-2023**

A: Absolute number of TTVA cases treated between 2018-2023; B: Incidence of total periprocedural complications among all TTVA patients; C: Frequency of periprocedural complications in CIED patients; D: Frequency of periprocedural complications in non-CIED patients. The composite of any complications included RCA stenting, cardiac injury requiring pericardiocentesis or emergency cardiac surgery, and device detachment.

**Supplemental Tables and supporting information**

| **Supplemental Table 1: CIED-related variables** | | | |
| --- | --- | --- | --- |
|  | CIED patients  (n=41) |  |  |
| Device type |  |  |  |
| Single-chamber PM | 36.59 % (15/41) |  |  |
| + ICD | 6.67 % (1/15) |  |  |
| Dual-chamber PM | 46.34 % (19/41) |  |  |
| CRT | 17.07 % (7/41) |  |  |
| + ICD | 42.86 %(3/7) |  |  |
| Age of CIED generator (months) | 94.57 ± 231.63 |  |  |
| Indication for PM |  |  |  |
| Bradyarrhythmias | 26.82 % (11/41) |  |  |
| Ischemic cardiomyopathy | 7.32 % (3/41) |  |  |
| Dilated cardiomyopathy | 4.88 % (2/41) |  |  |
| 3rd degree AV-Block | 17.07 % (7/41) |  |  |
| Sick Sinus Syndrome | 14.63 % (6/41) |  |  |
| Unknown | 29.27 % (12/41) |  |  |
| RV-lead position  in relation to Tricuspid valve |  |  |  |
| Central | 43.9 % (18/41) |  |  |
| Anteroposterior | 7.32 % (3/41) |  |  |
| Anteroseptal | 4.88 % (2/41) |  |  |
| Posteroseptal | 36.59 % (15/41) |  |  |
| Multiple leads | 4.88% (2/41) |  |  |
| Other | 2.44 % (1/41) |  |  |
| Values are presented in Percent (%) or as Mean ± Standard deviation (SD);  PM. Pacemaker; AICD. Automated implantable cardioverter defibrillator; CRT. Cardiac resynchronization therapy; RV. Right ventricle; RA. Right atrium; LV. Left ventricle | | | |

| **Supplemental Table 2: Baseline echocardiographic variables in CIED and non-CIED patients stratified by intraprocedural success (TVARC)** | | | | | |  |
| --- | --- | --- | --- | --- | --- | --- |
|  | CIED patients (n=41) | | | Non CIED patients (n=41) | | |
|  | No IS TVARC  (n=13) | IS TVARC  (n=28) | p value | No IS TVARC  (n=48) | IS TVARC  (n=115) | p value |
| LVEF (%) | 56.08 ± 6.32 | 49.79 ± 12.31 | **0.037#** | 57.84 ± 8.29 | 55.25 ± 8.944 | 0.086 |
| RV basal diameter (mm) | 52.86 ± 10.18 | 44.1 ± 10.14 | **0.014** | 51.3 ± 6.67 | 45.59 ± 7.98 | **<0.001** |
| RA area (cm^2^) | 41.19 ± 9.76 | 33.45 ± 6.66 | **0.024#** | 39.88 ± 12.46 | 32.88 ± 9.27 | **<0.001** |
| RV FAC (%) | 39.8 ± 7.02 | 43.91 ± 12.77 | 0.229# | 37.68 ± 11.12 | 41.63 ± 10.79 | **0.042** |
| TR EROA (cm^2^) | 1.26 ± 0.78 | 0.73 ± 0.36 | **0.041#** | 0.91 ± 0.35 | 0.66 ± 0.28 | **<0.001** |
| TR VC (mm) | 20.17 ± 3.51 | 13.38 ± 5.15 | **<0.001** | 16.84 ± 4.86 | 13.16 ± 5.1 | **<0.001** |
| TR regurgitation volume (ml) | 74.64 ± 36.72 | 55.44 ± 29.92 | 0.107 | 65.12 ± 27.87 | 54.82 ± 22.46 | **0.020** |
| Anteroseptal Annulus diameter (mm) | 47.17 ± 4.06 | 41.82 ± 3.92 | **<0.001** | 45.40 ± 6.47 | 41.23 ± 5.89 | **<0.001** |
| IVCd (mm) | 26 ± 9.59 | 25.35 ± 6.28 | 0.817 | 26.79 ± 7.13 | 23.54 ± 6.51 | **0.008** |
| TRI-Score (%) | 18.62 ± 13.04 | 26.29 ± 18.14 | 0.090 | 26.98 ± 21.28 | 17.46 ± 12.80 | **0.005#** |
| Lead impingement | 21.43 % (6/28) | 30.77 % (4/13) | 0.698* |  |  |  |
| Values are presented in Percent % or as Mean ± SD. Standard deviation;  CRT. Cardiac resynchronization therapy; IS: intraprocedural success; ICD. Implantable cardioverter defibrillator; RV. Right ventricle; RA. Right atrium; LV. Left ventricle; #Equal variance not assumed after Levene`s Test for Equality of Variance | | | | | | |

| **Supplemental Table 3: Overall echocardiographic efficacy endpoints** | | | |
| --- | --- | --- | --- |
|  | Baseline  (n=157) | 30-day follow-up  (n=157) | p-value |
| **Echocardiographic efficacy endpoints** |  |  |  |
| LVEF (%) | 55.4 ± 9.34 | 56.11 ± 8.89 | 0.156 |
| RV basal diameter (mm) | 47.3 ± 7.1 | 41.67 ± 7.34 | **<0.0001****** |
| RV fractional area change (FAC) (%) | 40.68 ± 10.82 | 37.63 ± 10.79 | **0.008**** |
| RA area (cm2) | 35.41 ± 9.94 | 29.41 ± 9.63 | **<0.0001****** |
| TR effective regurgitation orifice area (EROA) (cm2) | 0.77 ± 0.35 | 0.39 ± 0.9 | **<0.0001****** |
| TR vena contracta (mm) | 14.54 ± 5.23 | 6.82 ± 4.42 | **<0.0001****** |
| TR regurgitation volume (ml) | 56.97 ± 23.64 | 25.73 ± 20.01 | **<0.0001****** |
| Systolic pulmonary artery pressure (sysPAP) (mmHg) | 38.19 ± 13.7 | 44.84 ± 15.71 | **<0.0001****** |
| Inferior vena cava diameter (IVCd) (mm) | 24.6 ± 6.75 | 20.5 ± 5.82 | **<0.0001****** |
| Values are presented in Percent (%), Mean ± Standard deviation (SD), or Median* ± interquartile range (IQR);  RV. Right ventricle; RA. Right atrium; BL. Baseline. FU. Follow-up (mean 73 +/- 41 days); Δ. Delta (difference between two time points) | | | |
